# Antibiotic rotation strategies to reduce antimicrobial resistance in Gram-negative bacteria in European intensive care units: study protocol for a cluster-randomized crossover controlled trial

**DOI:** 10.1186/1745-6215-15-277

**Published:** 2014-07-10

**Authors:** Pleun J van Duijn, Marc JM Bonten

**Affiliations:** 1 Julius Center for Health Sciences and Primary Care, University Medical Center Utrecht, Universiteitsweg 100, CG 3584, Utrecht, The Netherlands; 2 Department of Clinical Microbiology, Molecular Epidemiology of Infectious Disease, University Medical Center Utrecht, Heidelberglaan 100, CX 3584, Utrecht, The Netherlands

## Abstract

**Background:**

Intensive care units (ICU) are epicenters for the emergence of antibiotic-resistant Gram-negative bacteria (ARGNB) because of high rates of antibiotic usage, rapid patient turnover, immunological susceptibility of acutely ill patients, and frequent contact between healthcare workers and patients, facilitating cross-transmission.

Antibiotic stewardship programs are considered important to reduce antibiotic resistance, but the effectiveness of strategies such as, for instance, antibiotic rotation, have not been determined rigorously. Interpretation of available studies on antibiotic rotation is hampered by heterogeneity in implemented strategies and suboptimal study designs. In this cluster-randomized, crossover trial the effects of two antibiotic rotation strategies, antibiotic mixing and cycling, on the prevalence of ARGNB in ICUs are determined. Antibiotic mixing aims to create maximum antibiotic heterogeneity, and cycling aims to create maximum antibiotic homogeneity during consecutive periods.

**Methods/Design:**

This is an open cluster-randomized crossover study of mixing and cycling of antibiotics in eight ICUs in five European countries. During cycling (9 months) third- or fourth-generation cephalosporins, piperacillin-tazobactam and carbapenems will be rotated during consecutive 6-week periods as the primary empiric treatment in patients suspected of infection caused by Gram-negative bacteria. During mixing (9 months), the same antibiotics will be rotated for each consecutive antibiotic course. Both intervention periods will be preceded by a baseline period of 4 months. ICUs will be randomized to consecutively implement either the mixing and then cycling strategy, or vice versa. The primary outcome is the ICU prevalence of ARGNB, determined through monthly point-prevalence screening of oropharynx and perineum. Secondary outcomes are rates of acquisition of ARGNB, bacteremia and appropriateness of therapy, length of stay in the ICU and ICU mortality. Results will be adjusted for intracluster correlation, and patient- and ICU-level variables of case-mix and infection-prevention measures using advanced regression modeling.

**Discussion:**

This trial will determine the effects of antibiotic mixing and cycling on the unit-wide prevalence of ARGNB in ICUs.

**Trial registration:**

ClinicalTrials.gov NCT01293071 December 2010.

## Background

Infections caused by Gram-negative bacteria frequently complicate treatment of critically ill patients in the intensive care unit (ICU). Such ICU-acquired infections are associated with higher morbidity and mortality [1]. The severity of illness in these patients often precludes awaiting diagnostic microbiology results. Treatment is therefore mostly empiric, covering a broad range of potential pathogens, increasing selective pressure for antibiotic-resistant bacteria.

As a response, antibiotic stewardship programs aim to optimize the rational and prudent use of antibiotics. These programs attempt to reduce selective pressure by reducing overall antibiotic consumption, but also to optimize the choice of the antibiotic, dosing and administration route. As such, antibiotic rotation has been proposed to reduce antibiotic resistance through systematically rotating antibiotics or antibiotic classes for empirical treatment.

There are two types of rotation schemes: mixing and cycling. In mixing, the treatment is changed with every new antibiotic course, and in cycling, empiric antibiotics change per time block (weeks or months). These interventions have been evaluated in ICUs, but also in neonatal-, pediatric-, oncology- and cardiothoracic-surgery departments [2-23]. The methodology of these studies and the results obtained, however, vary widely. Importantly, study design, data collection and statistical analyses did not always take into account clustering of antibiotic resistance within ICUs, confounding by antibiotic use and changes in case-mix or infection prevention measures [24].

This multicenter cluster randomized crossover trial was designed to determine the effects of mixing and cycling of antibiotics in eight European ICUs, incorporating the most relevant confounders and adjusting for clustering of results in the analysis. For uniformity of reporting, we have used the CONSORT 2010 statement: extension to cluster randomized trials [25].

### Study objectives

The primary objective of this trial is to compare the effects of a strategy of antibiotic mixing (rotation of empirical antibiotic treatment per next individual patient) to a strategy of antibiotic cycling (preferred empirical antibiotic treatment changes every 6 weeks) on the mean unit-wide prevalence of antibiotic-resistant Gram-negative bacteria (ARGNB). The primary hypothesis is superiority of one intervention arm over the other.

## Methods/Design

### Study design

**Figure 1:**
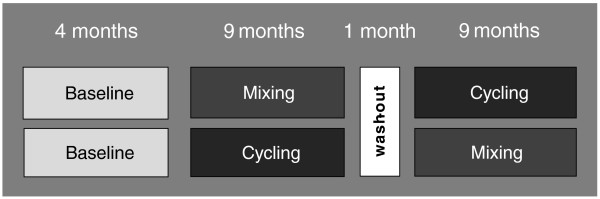
**All 8 intensive care units (ICUs) start with 4 months standard care and then perform both interventions: cycling then mixing or vice versa.** ICUs are randomized to start with either cycling or mixing. After 9 months of intervention and a 1 month standard care wash-out period, the ICUs cross over into the second intervention for the final 9 months.

The trial has a cluster-randomized, crossover design. All ICUs start with a 4-month standard care period with no interventions and are then randomized to one of two interventions of 9 months. After a wash-out period of 1 month, the ICUs cross over and perform the alternate rotation strategy for a second 9-month period (Figure 1). Inclusions will start January 2011 and the last ICU will finish February 2014.

### Participants

The participating ICUs are the primary object of study, individual data of all admitted patients will be used for secondary endpoints. Inclusion criteria for ICUs are listed in the “Intensive care unit inclusion criteria” section. ICUs have been selected through a tendering procedure according to EU regulations. An open tender communiqué was sent to hospitals directly and published on public websites of the European Society of Clinical Microbiology and Infectious Diseases and the European Society of Intensive Care Medicine. Interested ICUs were screened through questionnaires and on-site visits to assess eligibility. Thirty-eight ICUs showed an interest, of which eight ICUs in five countries were selected (in Belgium, France (n = 2), Germany (n = 2), Portugal and Slovenia (n = 2)). For all ICUs, IRB approval that required a waiver for individual patient written informed consent was obtained. The SATURN ICU trial was registered in the ClinicalTrials.gov (NCT01293071).

### Intensive care unit inclusion criteria

#### Inclusion criteria

1. At least eight beds with capacity of mechanical ventilation

2. Presence of at least one research nurse (or equivalent personnel) dedicated to the trial

3. Facilities for storage of screening swabs at -70 degrees Celsius

4. Approval of study protocol by the local Institutional Review Board (IRB)

5. Ability to obtain an informed consent waiver for individual patients

6. Ability to obtain written consent by physician and nursing staff representative to participate in the trial

7. Availability of a digital data patient management system for data extraction

8. No planned implementation of other interventions that may affect resistance prevalence during the study period

9. Not being an Intensive Care Burn Unit, Cardiothoracic Surgery Unit, Pediatric Intensive Care Unit, or Neonatal Intensive Care Unit

### Interventions

During the intervention periods, the ICU-wide preferred empirical treatment for ICU-acquired infections with Gram-negative bacteria is rotated according to two protocols; mixing and cycling. The three rotated antibiotics will be 1) third- or fourth- generation cephalosporins, 2) piperacillin-tazobactam and 3) carbapenems.

During mixing, the preferred choice for empiric antimicrobial treatment changes with every newly prescribed empiric antibiotic course. During cycling, empiric treatment changes every 6 weeks. The order of interventions and the order of rotated antibiotics are randomized at the beginning of the trial by a person not involved in the design or execution of the study.

Treating physicians can deviate from study protocol at any point and for any reason because of patient safety or as part of de-escalation of antibiotic treatment. Combination therapy with preferred antibiotics (such as addition of an aminoglycoside or coverage of Gram-positive bacteria) is allowed. ICU-specific procedures related to standard hygienic measures, monitoring practices, outbreak management and any other infection prevention measures will not be dictated by study protocol.

Overall antibiotic use will be derived from ward-level consumption based on either individual prescription data or weekly unit level administrative antibiotic data.

As a proxy for protocol adherence, weekly point-prevalence measurements of antibiotic consumption will be taken. Antibiotic courses are then counted, regardless of dose or route of administration. The counts will then be translated to fractions of study-adherent antibiotics of all antibiotics. With optimal adherence, the three preferred antibiotics should be in equal proportions during mixing, whereas the preferred antibiotic should be dominant during cycling. Weekly graphs of ICU-specific results are then returned to each of the ICUs to provide compliance feedback.

### Outcomes

The primary endpoint is the mean ICU-level point-prevalence of ARGNB, which will be obtained by combining the nine monthly point-prevalence screening results for each study period. Unit-wide monthly point-prevalence surveys will be performed by obtaining swabs from oropharynx and perineum from all patients present in the ICU during the point-prevalence survey. Swabs will be frozen directly and analyzed in a central microbiology laboratory (Appendix). Resistance is defined as extended-spectrum beta-lactamase (ESBL)-producing Enterobacteriaceae, Piperacillin-Tazobactam resistance in Enterobacteriaceae, *Pseudomonas aeruginosa* and *Acinetobacter* species, and Carbapenem resistance in *P. aeruginosa* and *Acinetobacter* species (Table 1).

**Table 1:** Outcome resistance per species

**Species**	**Extended-spectrum beta-lactamase**	**Piperacillin-Tazobactam resistance**	**Carbapenem resistance**
	**(ESBL)-producing**		
Enterobacteriaceae	+	+	
*Pseudomonas aeruginosa*		+	+
*Acinetobacter* species		+	+

Secondary endpoints are: 1) individual patient acquisition rates of ARGNB per admission days at risk; defined as the first positive culture for ARGNB >48 hrs after admission. 2) ICU-acquired bacteremia rates with ARGNB per admission days at risk; 3) percentage of appropriate empirical treatment of ICU-acquired bacteremia; 4) mean length of stay in ICU; and 5) mean in-ICU-mortality.

For these secondary outcomes, clinical culture results will be used to determine ARGNB colonization status. Appropriateness of empirical treatment will be based on blood culture isolate antibiograms and antibiotic treatment prescribed. Length of ICU stay and in-ICU mortality will be derived from computerized patient records.

### Sample size calculation

Sample size calculations for individually randomized patients assume that patient outcome is independent of other patients’ outcomes, an assumption that is frequently violated when investigating the dynamics of infectious disease. Ignoring this interpatient dependency may lead to overestimation of treatment effect and underestimation of the necessary sample size.

The calculated sample size without clustering is 392 cultured patients per intervention arm, assuming a binomial distribution and based on 80% power to detect an absolute change of 10% in resistance prevalence with a 95% confidence level using a two-sided test. The sample size is based on a worst-case scenario with regard to precision; the widest distribution and thus largest needed sample size is around 50% for a binomial distribution. Therefore, the prevalence decrease was set from 55% to 45%. As an illustration, to detect a reduction from 30% to 20%, 291 patients per intervention period are needed. To include clustering effects we used an Intra-class Correlation Coefficient (ICC) of 0.01, based on a previous cluster-randomized ICU study [26]. For estimation of the average cluster sample size it was assumed that each ICU had 15 beds for every 9-month-prevalence measurement, resulting in 135 samples per ICU, per intervention arm, which yields a design effect of 2.35 [25]. Multiplying the unadjusted sample size of 392 with the design effect means 921 sampled patients for one arm, and that 1,842 for both intervention arms are needed.

With an expected 2,160 sampled patients for both intervention arms (2 times 9 point-prevalence measurements in eight 15-bed ICUs), our study should therefore be adequately powered.

### Data collection

Dedicated staff will collect data from (digital) patient charts and microbiology reports. Screening swabs will be collected by qualified personnel, either by the ICU-staff or study-nurse. Data of potential confounders is collected from computerized individual patient records (for example, age, gender, admission diagnosis). Ward-specific confounder data (such as hand hygiene compliance, use of indwelling devices, use of isolation measures, and staffing ratios) are obtained from monthly point-prevalence measurements.

Weekly point-prevalence measurements of antibiotic consumption will be collected either directly on the ward or from the patient data management system.

### Analysis

Two types of outcome analysis will be performed: analysis of the ICU-level of resistance prevalence (primary outcome) and analyses on individual patient level, including the secondary outcome measures (Table 2).

**Table 2:** Summary of analyses for secondary outcomes

**Outcome**	**Analysis**
Acquisition rates of antibiotic resistant Gram-negative bacteria (ARGNB)	McNemar’s test; Cox proportional hazard regression with random effects for ICUs.
Intensive care unit (ICU)-acquired bacteremia rates	McNemar’s test; Cox proportional hazard regression with random effects for ICUs.
Percentage of appropriate empirical treatment of ICU-acquired bacteremia	McNemar’s test; Generalized linear regression with random effects for ICUs.
Mean length of stay in ICU	Paired t-test; Generalized linear regression with random effects for ICUs.
In-ICU mortality	McNemar’s test; Cox proportional hazard regression with random effects for ICUs.

Descriptive analysis will be performed of baseline characteristics and covariates. Where appropriate, bivariate tests will be performed. Bivariate statistical testing and advanced regression analysis will be used to model the effect of the interventions on resistance prevalence and acquisition. This will include adjusting, if necessary, for clustering of outcome data and confounding within ICUs by patient demographics, antibiotic consumption, illness severity, infection prevention measures and time trends. Results from point-prevalence measurement will be analyzed as continuous or count measurements per ICU for each intervention period, taking auto-correlation within ICUs into account in regression analysis.

The primary outcome measurements will be analyzed using McNemar’s test for dependent pairs with all outcomes pooled for mixing or cycling. Bivariate tests, however, do not take into account differences in effects between hospitals, trends over time or possible confounding. Therefore, a stepwise mixed effects model will be constructed using the prevalence of resistance colonization as outcome, and the interventions as a factor. To assess differences in treatment effects between ICUs and time trends over study periods, random effects for the eight ICUs and the nine longitudinal measurements per ICU per study period will be added stepwise to the model. In the case of an imbalanced case-mix between interventions, confounders can be added to the model to adjust for these differences. This will facilitate adjustment for intracluster correlation and for imbalances in the study arm case mix (patient and ICU characteristics) not caused by the interventions themselves.

For the secondary outcomes, analyses are stated in Table 2. For acquisition rates of ARGNB, bacteremia with resistant Gram-negative bacteria, and mortality, the McNemar’s test for dependent pairs will be performed first, followed by the Cox-proportional hazard regression models, accounting for differences in time-at-risk, inter-ICU intervention effect differences and possible cofounding. For the proportion of patients receiving appropriate empirical treatment, the same bivariate test and the same type regression model is used as for the primary outcome and by the same argumentation. Mean length of ICU-stay will be tested using the t-test for dependent means and if necessary with linear mixed effects regression, again with inclusion of random effects for individual ICUs and assessing possible confounding.

Because of the crossover design, period effects and carry-over effects will be assessed.

## Discussion

### Previous studies

The association between antibiotic use and selective pressure for ARGNB, together with the incomplete evidence base for antibiotic rotation strategies in ICU settings, warrants more research on this subject. The lack of a consistently applied methodology to study antibiotic rotation strategies, and the suboptimal designs applied in some studies, seriously hamper interpretation of available results at present [24]. We have addressed these issues in our current study, attempting to maximize protocol adherence and generalizability for European ICUs (Table 3). Nonetheless, this unavoidably led to compromises with regard to interventions, study design and analyses.

**Table 3:** Methodological characteristics and key points

**Study design feature**	**Advantages**	**Disadvantages**	**Remarks**
**Cluster allocation**	Prevents allocation bias	Susceptible to case mix fluctuations in time	Prevented by adequate intervention period length
	Prevents between-intervention correlation as compared with individual randomization	Creates cluster correlation of outcomes	Will be accounted for in analysis
**Open treatment design**	Transparency in patient treatment	Different treatment adherence between different preferred antibiotics	Does not differ between interventions
**Pre-intervention control period**	Enables comparison with standard care. Enables time trend analysis for time-dependent increase in prevalence	No control group parallel in time	Comparable parallel groups/intensive care units (ICUs) not available, are expected to have higher heterogeneity in ICU characteristics than within-ICU comparisons using a crossover design
**Crossover**	Intervention comparison within ICUs equals out differences that influence outcome	Increases trial time-span and effect of baseline resistance increases over time	Addressed with time-trend analysis using pre-intervention control period

### Selection of antibiotics, timing of interventions and outcome measures

We decided to target currently important pathogens, ARGNB, and the antibiotics mostly used for those infections. Consequently, the used antibiotic classes will be all beta-lactam antibiotics (in the case of piperacillin-tazobactam, in combinations with a beta-lactamase inhibitor). It could be hypothesized that rotation of antibiotics with different mechanisms of action would increase the effects of mixing and/or cycling. However, given the current increase in incidence of ESBL-producing bacteria and resistance to fluoroquinolones, choices of empiric antibiotics have become limited in the different regions of Europe. A too-specific choice of antibiotics would prevent inclusion of representative European ICUs, and would, therefore, reduce generalizability of findings. Indeed, the antibiotic strategies evaluated in this study may not be relevant for settings with much lower levels of ESBL-producing bacteria or in settings with endemicity of carbapenem-resistant Enterobacteriaceae.

The optimal timing of cycling interventions is as of yet unknown, and periods of weeks to months have been used in previous studies [2-18,20-22,27], and studies using mathematical modeling also did not reach definite conclusions as to which rotation timing is optimal [28-37]. The studied intervention types include cycling and mixing, but dynamic and hybrid interventions have also been proposed: using prevalence data when to switch and when to use either cycling or mixing [37].

We decided to use cycling periods of 6 weeks, to evaluate reintroduction of the intervention antibiotics. Six-week periods allow two complete cycles of three antibiotics in 9 months. The aim of this reintroduction is to investigate possibly faster re-emergence of antibiotic resistance during the second cycle of antibiotics.

The monthly ICU-level, point-prevalence measurements will provide unbiased data for ARGNB prevalence. Furthermore, clinical culture results provide additional specific, though less sensitive, colonization incidence data.

### Study design

The open cluster randomized design with a baseline period and crossover of interventions has methodological advantages but also limitations over the quasi-experimental before-after design or patient randomized studies.

First, the cluster design, with interventions applied unit-wide, prevents allocation bias within ICUs and reduces contamination of intervention effects. With individual patient randomization, different interventions will be executed in the same ICU at the same time. The transmissibility of infectious disease implies that colonization rates for patients in the same ward will become dependent, which may well decrease outcome differences between interventions. A cluster-randomized study design physically separates the two intervention populations and thereby prevents transmission-based dependency in resistance outcomes. Possible associations between resistance acquisition within an ICU are now associated with only one intervention. The disadvantage is the introduction of clustering, or correlation of results within ICUs, which needs to be accounted for in sample size calculations and analysis.

Due to the consecutive inclusions in cluster randomized studies, these are more susceptible to chance fluctuations potentially causing selection bias, which needs to be assessed and adjusted for, if present.

Second, the open design may lead to allocation bias, where certain patient groups will not be eligible for the treatment with the preferred study antibiotic. Hospital specific distributions of parameters such as treatment indication and/or comorbidities will therefore influence adherence and possibly the effect of interventions, depending on the intervention phase and preferred antibiotic. The crossover design, however, will prevent differential distribution/bias between intervention groups. Nevertheless, the different interventions could have implications for adherence still causing bias between interventions with regard to intervention effect. Assessing differences in intervention adherence is therefore part of the analysis.

Third, both baseline antibiotic use -and resistance prevalence are known to change over time, even without interventions such as antibiotic rotation. Baseline study periods allow quantification of the effects of both interventions on overall antibiotic use and resistance prevalence, and extend the possibilities for time-trend analysis, given the absence of an adequate parallel control group in time. It was decided not to include such a parallel control group because of the large differences of patient populations between ICUs, even within the same country.

Fourth, the crossover of interventions provides adjustment for differences between units that may also affect the prevalence of ARGNB.

Finally, with regard to outcome data collection, centralized processing of samples will prevent hospital specific differences in detection of resistance. Admission and discharge swabs are not obtained, making this study less suited to determine acquisition of cross-transmission rates of ARGNB.

### Sample size and statistical analysis

To our knowledge, this will be the first multicenter study with eight ICUs on these interventions aiming to include approximately 10,000 patients in five different countries [38]. The power calculation included adjustment for intracluster correlation and provides precision, accuracy and external validity.

The intraclass correlation coefficient (ICC) was used based on mortality data from a multicenter study in 13 Dutch ICUs. The ICC could therefore be different for antibiotic resistance data, which would change the power of our sample size. Nonetheless, with our current estimated sample size, the ICC could increase 60% to 0.016. In addition, this calculation does not account for the crossover design, ignoring the reduction in interclass correlation resulting from this design.

Also, the sample-size calculation assumed a similar ICC for all ICUs, did not take clustering of results obtained on the same sampling day into account, and the ICC did not include a reported standard error, as recommended in the CONSORT statement.

Outlining the analysis plan, descriptive analysis and exploration of covariates will precede final regression analysis. In general terms, the prevalence during both interventions and secondary outcomes will be compared, assessing whether any - and which of the two interventions - is superior over the other. The baseline periods will be used to compare standard care to the intervention periods. The predefined analysis will include bivariate testing and stepwise methods adjusting for possible influences of the study design and time effects. This includes clustering of outcome data, time trends and confounding using Generalized Linear Mixed Models. Secondary outcomes will be analyzed using bivariate testing, and Cox-proportional hazard models also accounting for random effects.

## Conclusion

Better understanding of the associations between antibiotic pressure and resistance emergence is needed and important. This trial will provide further insight in the use of mixing and cycling of antibiotics and guide future practice guidelines and clinical and mathematical modeling studies on the effects of antibiotic policies.

## Trial status

Recruiting.

## Appendix

### Screening swab protocol

Screening swabs from monthly point-prevalence measurements will be taken from oropharynx and perineum of all patients in the ICU at that time point. Swabs will be put directly in medium and frozen at -70° Celsius. The swabs will then be shipped in batches under frozen conditions to the UMCU laboratory and centrally typed in the UMCU laboratory by dedicated laboratory analysts. Swabs will be cultured on five different plates (Table 4). Plates will be cultured overnight at 37 degrees Celsius, and morphologically distinct colonies will be selected and typed with the MALDI-TOF Mass Spectrometry. Resistance typing is performed with the Phoenix™ Automated Microbiology System. ESBL positive isolates will be sent to University of Antwerp (UA), Belgium for confirmation of ESBL-genes. *Acinetobacter*, *Pseudomonas* and *Stenotrophomonas* species will be sent to the Barcelona Centre for International Health Research (CRESIB), Barcelona, Spain for pheno- and genotyping.

**Table 4:** Selective media plates

**Plates**	**Antibiotic (concentration)**
MacConkey	None
Extended-spectrum beta-lactamase (ESBL)	Oxoid Brilliance™ ESBL Agar
MacConkey/Ceftriaxone	Ceftriaxone (0.5 mg/L)
MacConkey/Pip-Tazo	Piperacillin-Tazobactam (4 mg/L)
MacConkey/Carbapenem	Meropenem (0.125 mg/L)

## Abbreviations

ARGNB: antibiotic-resistant Gram-negative bacteria; CONSORT: Consolidated Standards of Reporting Trials; ESBL: extended-spectrum beta-lactamase; ICC: intra-class correlation coefficient; ICU: intensive care unit; IRB: institutional review board.

## Competing interests

The authors declare that they have no competing interests.

## Authors’ contributions

PJD prepared the study protocol and this manuscript. MJMB was co-applicant for a European Union Framework Program 7 budget for this trial as part of the SATURN consortium, and also conceptualized the protocol and supervised preparation of the study protocol and manuscript. All authors read and approved the final manuscript.
